# Serum metabolomic and lipidomic profiling identifies diagnostic biomarkers for seropositive and seronegative rheumatoid arthritis patients

**DOI:** 10.1186/s12967-021-03169-7

**Published:** 2021-12-07

**Authors:** Hemi Luan, Wanjian Gu, Hua Li, Zi Wang, Lu Lu, Mengying Ke, Jiawei Lu, Wenjun Chen, Zhangzhang Lan, Yanlin Xiao, Jinyue Xu, Yi Zhang, Zongwei Cai, Shijia Liu, Wenyong Zhang

**Affiliations:** 1 School of Medicine, Academy for Advanced Interdisciplinary Studies, Southern University of Science and Technology, 1088 Xueyuan Rd., Shenzhen, China; 2 Affiliated Hospital of Nanjing University of Chinese Medicine, Nanjing, 210029 Jiangsu China; 3 Sustech Core Research Facilities, Southern University of Science and Technology, Shenzhen, China; 4 College of Pharmacy, Jiangsu Collaborative Innovation Center of Chinese Medicinal Resources Industrialization, Nanjing University of Chinese Medicine, Nanjing, 210046 China; 5 State Key Laboratory of Natural Medicines, School of Traditional Chinese Pharmacy, China Pharmaceutical University, Nanjing, 210009 China; 6 State Key Laboratory of Environmental and Biological Analysis (SKLEBA), Department of Chemistry, Hong Kong Baptist University, Kowloon Tong, Hong Kong, China

**Keywords:** Rheumatoid arthritis, Seronegative, Metabolomic, Lipidomic

## Abstract

**Background:**

Diagnosing seronegative rheumatoid arthritis (RA) can be challenging due to complex diagnostic criteria. We sought to discover diagnostic biomarkers for seronegative RA cases by studying metabolomic and lipidomic changes in RA patient serum.

**Methods:**

We performed comprehensive metabolomic and lipidomic profiling in serum of 225 RA patients and 100 normal controls. These samples were divided into a discovery set (n = 243) and a validation set (n = 82). A machine-learning-based multivariate classification model was constructed using distinctive metabolites and lipids signals.

**Results:**

Twenty-six metabolites and lipids were identified from the discovery cohort to construct a RA diagnosis model. The model was subsequently tested on a validation set and achieved accuracy of 90.2%, with sensitivity of 89.7% and specificity of 90.6%. Both seropositive and seronegative patients were identified using this model. A co-occurrence network using serum omics profiles was built and parsed into six modules, showing significant association between the inflammation and immune activity markers and aberrant metabolism of energy metabolism, lipids metabolism and amino acid metabolism. Acyl carnitines (20:3), aspartyl-phenylalanine, pipecolic acid, phosphatidylethanolamine PE (18:1) and lysophosphatidylethanolamine LPE (20:3) were positively correlated with the RA disease activity, while histidine and phosphatidic acid PA (28:0) were negatively correlated with the RA disease activity.

**Conclusions:**

A panel of 26 serum markers were selected from omics profiles to build a machine-learning-based prediction model that could aid in diagnosing seronegative RA patients. Potential markers were also identified in stratifying RA cases based on disease activity.

**Supplementary Information:**

The online version contains supplementary material available at 10.1186/s12967-021-03169-7.

## Introduction

Rheumatoid arthritis (RA) is a complex chronic autoimmune disease with variable presenting symptoms and serum autoantibody test results. Currently diagnosing RA is primarily based on clinical symptoms and the presence of various serum autoantibodies including rheumatoid factor (RF) and anti-citrullinated protein antibody (ACPA) [1–3]. Although most patients with confirmed RA have an abnormal test for RF and/ or ACPA, about 15%–20% of cases do not have the elevated levels of RF and ACPA [4]. Early recognition and assessment of the progression of RA are of paramount importance to avoid the irreversible damage. Therefore, developing reliable diagnostic biomarkers for evaluating seronegative RA is needed [1].

Chronic inflammatory state in RA causes metabolic changes that can serve as biomarkers for diagnosis, disease activity and treatment efficacy monitoring [5, 6]. The liquid chromatography–mass spectrometry (LC–MS)-based metabolomic and lipidomic approach is a powerful tool to discover potential biomarkers and metabolic pathway remodeling occurred in chronic diseases including RA [4, 7, 8]. However, small sample size and inadequate number of targets studied so far limit definitive conclusions drawing [4, 9–13]. The potential of using LC–MS-based metabolomic and lipidomic profiling for novel biomarker discovery to achieve more accurate diagnosis and disease activity monitoring remains largely unexplored.

In our study, we aimed to build a classification model that can be applied to both seropositive and seronegative patients by integrative LC–MS-based metabolomic and lipidomic profiling of serum samples from a large cohort of RA patients and normal control subjects.

## Materials and methods

### Clinical samples collection

Serum samples were collected from 225 RA patients and 100 normal control subjects at the Affiliated Hospital of Nanjing University of Chinese Medicine. The clinical and demographic characteristics of the study were summarized in Table 1. Disease activity for RA patients was assessed at the time of serum collection through quantification of tender and swollen joints, erythrocyte sedimentation rate (ESR), C-reactive protein (CRP) levels and the visual analog scale-grading of the patients. Cytokine concentrations were determined by immunoassay. Subjects in any one or more of the following categories were excluded from our analysis: (1) the presence of type I or II diabetes; (2) active viral and/or bacterial infection; (3) the presence of osteoarthritis. The study was approved by the medical ethics committee of the Affiliated Hospital of Nanjing University of Chinese Medicine and followed the tenets of the Declaration of Helsinki (2018NL-106-02). Written informed consents were obtained from all study subjects. Patients were clinically diagnosed with RA according to the American College of Rheumatology and European League Against Rheumatism (EULAR) 2010 criteria [9, 14]. Venous blood was collected in the morning before breakfast from all the participants, and then sera were separated and stored at − 80 °C until use.

**Table 1:** Clinical and demographic characteristics of the study participants

	Normal controls (n = 100)	RA (n = 225)	p value
Female: Male	69:31	189:36	
Age (years)	44.32 (21–66)	57.71 (27–87)	< 0.001
CRP (IU/mL)	2.6 (0.5–6.0)	19.3 (1.0–127.0)	0.003
ESR (mm/h)	9.7 (2.0–24.0)	43.8 (2.0–140.0)	0.002
IgA (IU/mL)	2.1 (0.9–3.9)	2.6 (0.2–6.4)	0.16
IgG (IU/mL)	12.8 (9.4–19.8)	14.2 (5.9–50.7)	0.39
IgM (IU/mL)	1.1 (0.4–3.2)	1.3 (0.3–4.4)	0.61
Disease duration (minimum–maximum in months)	–	9.7 (0.4–40)	
DAS28-CRP (minimum–maximum)	–	3.5 (1.0–7.1)	
DAS28-ESR (minimum–maximum)	–	4.0 (1.2–7.8)	
Rheumatoid factor positive	–	57.8%	
ACPA positive	–	42.2%	
ANA positive	–	49.4%	
AKA positive	–	49.4%	

Statistical significance was determined using unpaired two-tailed Student’s t test

ESR, Erythrocyte sedimentation rate; CRP, C-Reactive protein; IgA, Immunoglobulin A; IgG, Immunoglobulin G; IgM, Immunoglobulin M; ACPA, anti-citrullinated protein antibody; ANA, anti-nuclear antibodies; ANA, anti-keratin antibodies; DAS28-CRP, Disease activity score 28-joint count C reactive protein; DAS28-ESR, Disease activity score 28-joint count erythrocyte sedimentation rate

### Sample preparation

The metabolites and lipids in serum were isolated and treated as previously reported [15] with slight modifications. Fifty microliters of thawed serum samples were precipitated by adding 200 μl of cold acetonitrile. After centrifugation at 14,000*g* for 10 min at 4 °C, the supernatant was divided into four fractions: two for polar metabolites analysis by hydrophilic interaction liquid chromatography (HILIC)-LC–MS methods with positive ion mode electrospray ionization (ESI) and negative ion mode ESI, namely method 1 and method 2, respectively; two for lipids analysis by reverse phases (RP)-LC–MS methods with positive ion mode ESI and negative ion mode ESI, namely method 3 and method 4, respectively. After centrifugation, samples were dried and stored at − 80 °C until use. The quality control (QC) samples were prepared by mixing equal volumes of sera from RA patients and controls before sample preparation as they were aliquoted for analysis. These QC samples were utilized to estimate a “mean” profile representing all analytes encountered during the LC–MS analysis [16].

### Metabolomic and lipidomic analysis

For the polar metabolite profiling, dried samples were reconstituted in 100 μl of 80% methanol and analyzed by using a Dionex U3000 LC system coupled online to a Q Exactive Orbitrap MS instrument (Thermo Fisher Scientific, MA, USA) set at 35,000 resolution (at m/z 200). For the lipids profiling, dried samples were reconstituted in 100 μl of acetonitrile for instrument analysis. (HILIC)-LC–MS and (RP)-LC–MS were performed for metabolomic and lipidomic profiling, respectively. The details of the analytical experiments were described in Supplemental Materials. Data pre-processing was carried out as previously reported [17]. The XCMS package [18] was used for the extraction of peak abundances of metabolites and lipids. The background noises and contaminants in the peak tables were filtered by using CPVA with default parameters [19]. Quality assurance was achieved by the statTarget package [20] as follows. Briefly, peaks with more than 50% missing values were removed. The intensity of remaining peaks in samples was corrected according to the QC-RFSC algorithm. Principal component analysis (PCA)-based data quality evaluation was provided in Additional file 1: Fig. S1. Polar metabolites identification was according to the MS/MS spectra, the retention time of commercially available standard compounds, and the accurate mass of compounds from the Human Metabolome Database (www.hmdb.ca) and MassBank of North America (http://mona.fiehnlab.ucdavis.edu). Lipids were identified based on MS/MS match by using the LipidSearch software v4.1 (Thermo Fisher Scientific, CA, USA). The parameters in LipidSearch were set as follows: precursor tolerance at 5 ppm, product tolerance at 5 ppm, product ion threshold at 5%, and intensity threshold at 1.0%. Quantitation and Toprank filter were turned on. Main node filters were set to Main Isomer Peaks, and ID quality was graded from A-B. Pathway analysis based on identified metabolites was carried out using Metaboanalyst 4.0 [21] according to the Kyoto Encyclopedia of Genes and Genomes (KEGG) pathway database (www.genome.jp/kegg/).

### Data analyses

The Mann–Whitney–Wilcoxon test or Welch’s t-test, was performed to measure the significance of each peak, with results adjusted for multiple testing using false discovery rates (FDR) correction. The cross-validated partial least squares discriminant analysis (PLS-DA) and variable importance in the projection (VIP_plsda_) were calculated using the statTarget package. Serum metabolites passing the threshold of VIP_plsda_ > 1, fold change > 1.2 or < 0.8, and adjusted p-value < 0.05 were regarded as potential markers between the RA and NCs groups. The alluvial plots and forest plot were performed using the ggalluvial package and forestplot package, respectively. The co-occurrence network was carried out and visualized in the igraph package (https://igraph.org). Spearman’s rank correlation was used for a measure of correlation between two variables. The nodes represent clinical parameters or metabolites and lipids, and two nodes were connected if they were significantly correlated (adjusted p-value < 0.05 and r-value > 0.2). The ordinal regression was performed by using the ordinalgmifs package for fitting an ordinal response model [22]. Coupling the receiver operating characteristic curve (ROC) with its area under the curve (AUC), a widely used method to estimate the diagnostic potential of a classifier in clinical applications, was performed using the pROC package [23]. All packages were implemented using the freely available R language.

## Results

### Clinical data and patient characteristics

**Fig. 1:**
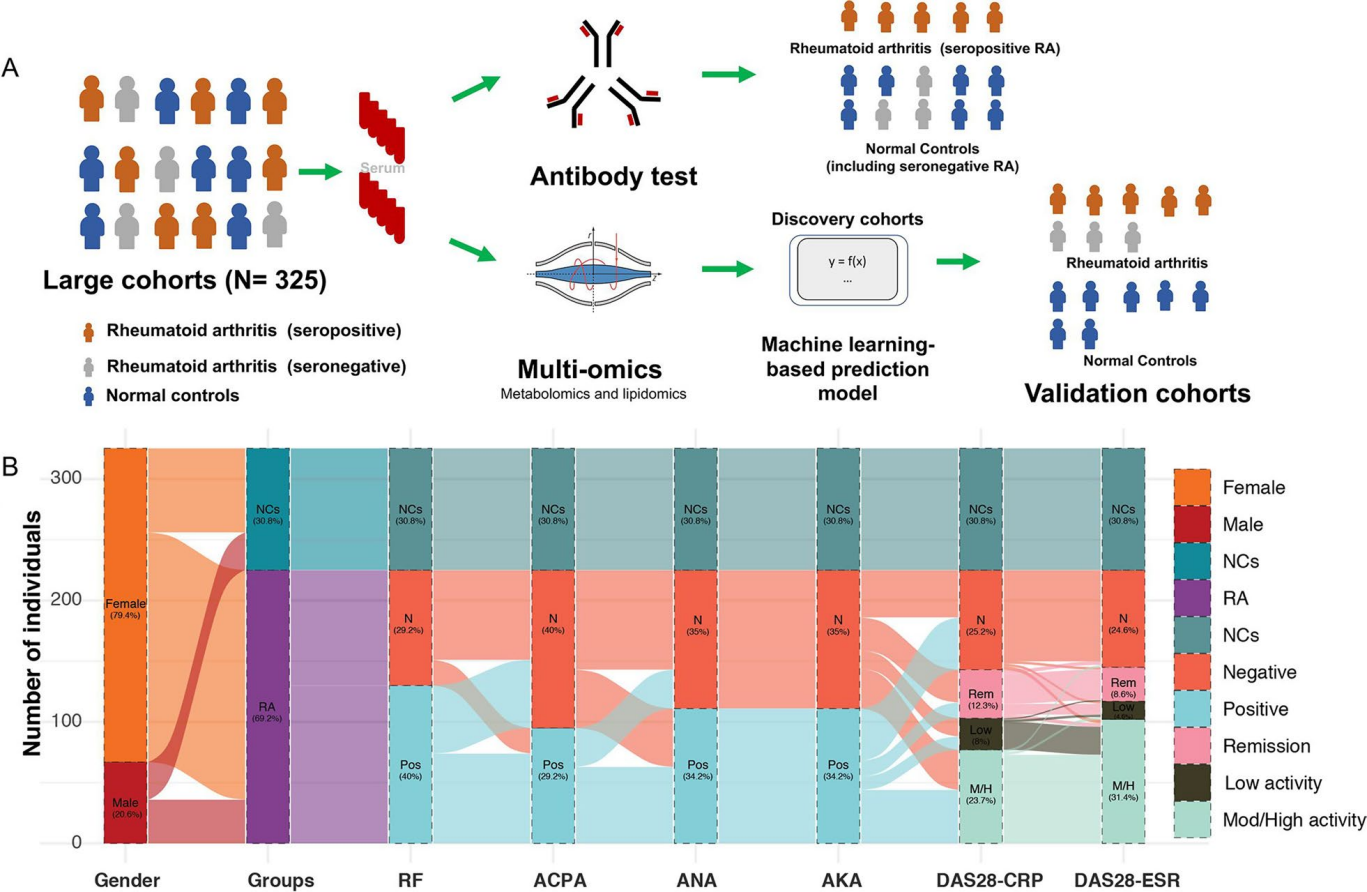
A Study design and clinical outcomes. **A** Schematic overview of the study cohort and the methods description. **B** Alluvial plot showing the number of individuals crossing over the disease groups, gender, DAS28-CRP, DAS28-ESR, RA-related autoantibodies such as rheumatoid factor (RF), anti-citrullinated protein antibody (ACPA), anti-nuclear antibodies (ANA), and anti-keratin antibodies (AKA)

The demographic information of the study participants was summarized in Table 1. Among 225 RA patients, the positive rate of RF and ACPA was 57.8% and 42.2%, respectively. These samples were randomly divided into two independent cohorts. The discovery set consisted of 172 RA patients and 71 normal controls (NCs), and the validation set consisted of 53 RA patients and 29 NCs (Fig. 1A). The alluvial plot shown in Fig. 1B displayed the number of individuals across the disease status, gender, disease activity score (DAS28-CRP and DAS28-ESR), as well as the status of autoantibodies such as rheumatoid factor (RF), ACPA, anti-nuclear antibodies (ANA), and anti-keratin antibodies (AKA). The positive rates of RF, ACPA, ANA, AKA in the RA patients were 57.8%, 42.2%, 49.4% and 49.4% (Table 1 and Fig. 1B).

### Metabolomic and lipidomic profile (MLP)

In four datasets generated from four different LC–MS runs, there were 1697 and 1960 high-confident peaks identified for polar metabolites with method 1 and 2, and 4771 and 3973 high-confident peaks identified for lipids with method 3 and 4. From this 12401 high-confident peaks we identified 265 metabolites and lipids (Additional file 1: Table S1), including 38 organic acids, 10 amines, 37 amino acids, 5 nucleotides, 4 bile acids, 26 acyl-carnitines (AcCa), 118 glycerophospholipids, 9 sphingolipids and 2 glycerolipids. These metabolites represent enriched metabolic pathways involving carnitine synthesis, oxidation of branched-chain fatty acids, biotin metabolism, malate-aspartate shuttle, citric acid cycle, urea cycle, phenylalanine and tyrosine metabolism, phospholipid biosynthesis and histidine metabolism (Additional file 1: Fig. S2).

### Clustering of test samples using MLP and pathway enrichment analysis

**Fig. 2:**
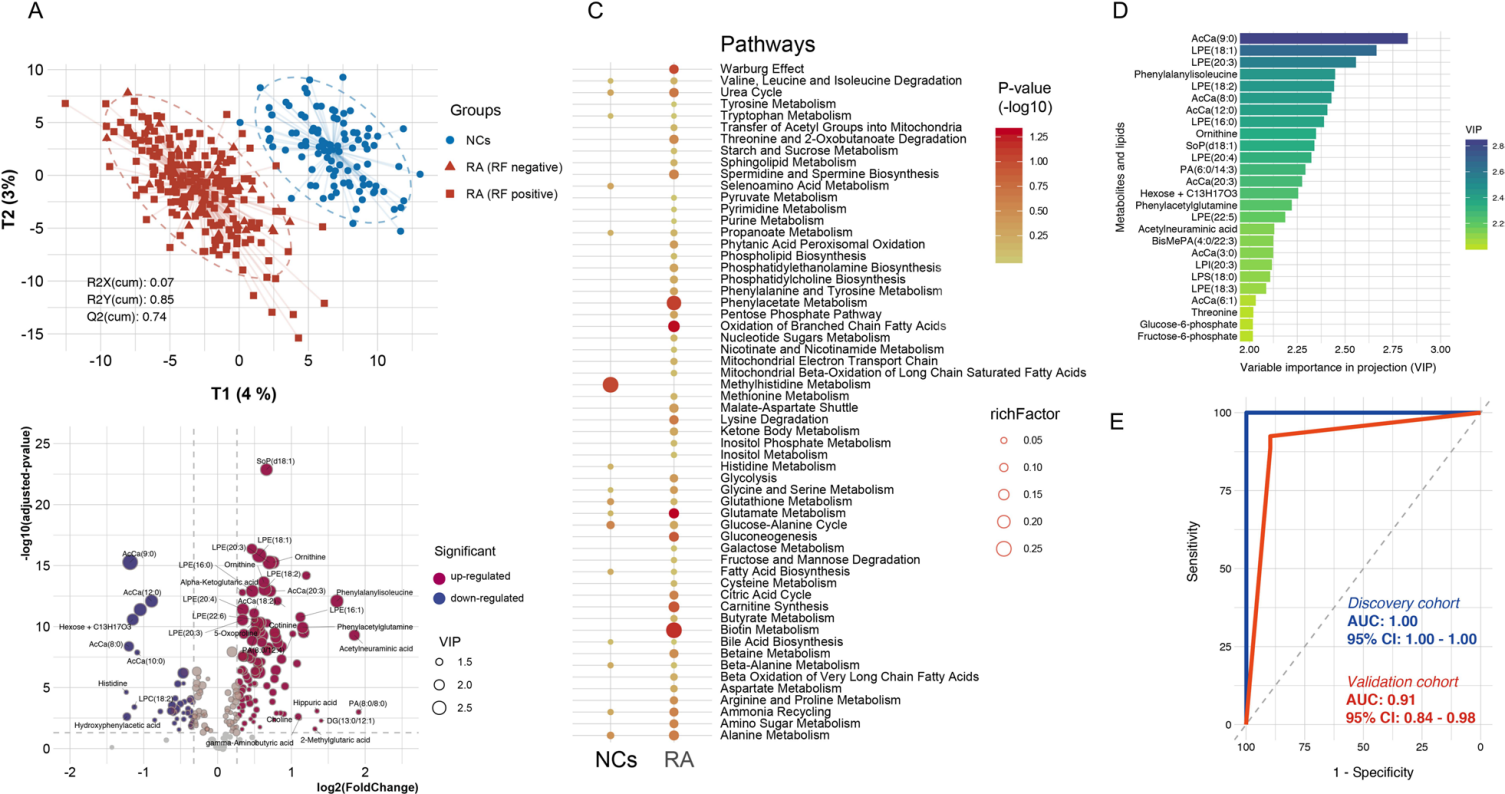
Metabolomic and lipidomic profiles and multivariate diagnostic model. **A** A PLS-DA model constructed from metabolomic and lipidomic profiling separated seropositive RA and seronegative RA patients from controls (NCs). **B** Volcano plot of metabolomic and lipidomic levels of RA versus NCs (x axis, fold change of RA to NCs; y axis, adjusted p value). Metabolites or lipids with VIP_plsda_ > 1, fold change > 1.2, adjusted p-value < 0.05 are colored in red and those with VIP_plsda_ > 1, fold change < 0.8, adjusted p-value < 0.05 in blue. **C** Metabolic pathway enrichment analysis of RA. **D** Metabolites and lipids with VIP_plsda_ > 2 selected for building a multivariate classification model. **E** ROC analysis of the multivariate classification model

To analyze whether any of the identified metabolites or lipids are associated with RA, we carried out the univariate and multivariate analysis of the identified metabolites and lipids. The well-established partial least squares-discriminant analysis (PLS-DA) demonstrated excellent separation of NCs from seropositive RA and seronegative RA based on MLP (Fig. 2A). The permutation tests (n = 1000) were performed to validate the PLS-DA model we used (Additional file 1: Fig. S3). This separation clearly demonstrated the difference in serum metabolite and lipid levels that existed between RA patients and normal control subjects. Statistical analysis identified 68 significantly upregulated and 38 downregulated serum metabolites and lipids that were correlated with RA (Fig. 2B). Pathway enrichment analysis (Fig. 2C) highlighted the top enriched metabolic pathways in RA. Specifically, metabolic products associated with Warburg effect, pentose phosphate metabolism, glycolysis, and lipid metabolism products involving phospholipid, sphingolipid, oxidation of branched chain fatty acids and carnitine synthesis were upregulated in the RA group, while histidine metabolism was downregulated.

### Constructing a multivariate classification model for RA

We constructed a multivariate classification model including 26 metabolites and lipids that could separate seropositive and seronegative RA cases from normal controls (Fig. 2D, E). We evaluated three machine learning algorithms such as binary logistic regression, random forest and support vector machine on the above-mentioned metabolites and lipids for the classification of RA cases using the discovery set (n = 243). The binary logistic regression algorithm based model trained with leave-one-out cross-validation had an accuracy of 100% (Fig. 2E, AUC = 1.00). This model was tested using the independent validation set (n = 82, Additional file 1: Table S2), and showed AUC = 0.91 with test sensitivity of 89.7% and specificity of 90.6% (Fig. 2E). We did an analysis of the 5 misclassified RA cases, and it is noteworthy that all of the 5 cases had borderline to positive RF antibody levels (Additional file 1: Table S3).

### Association of MLP and clinical parameters

**Fig. 3:**
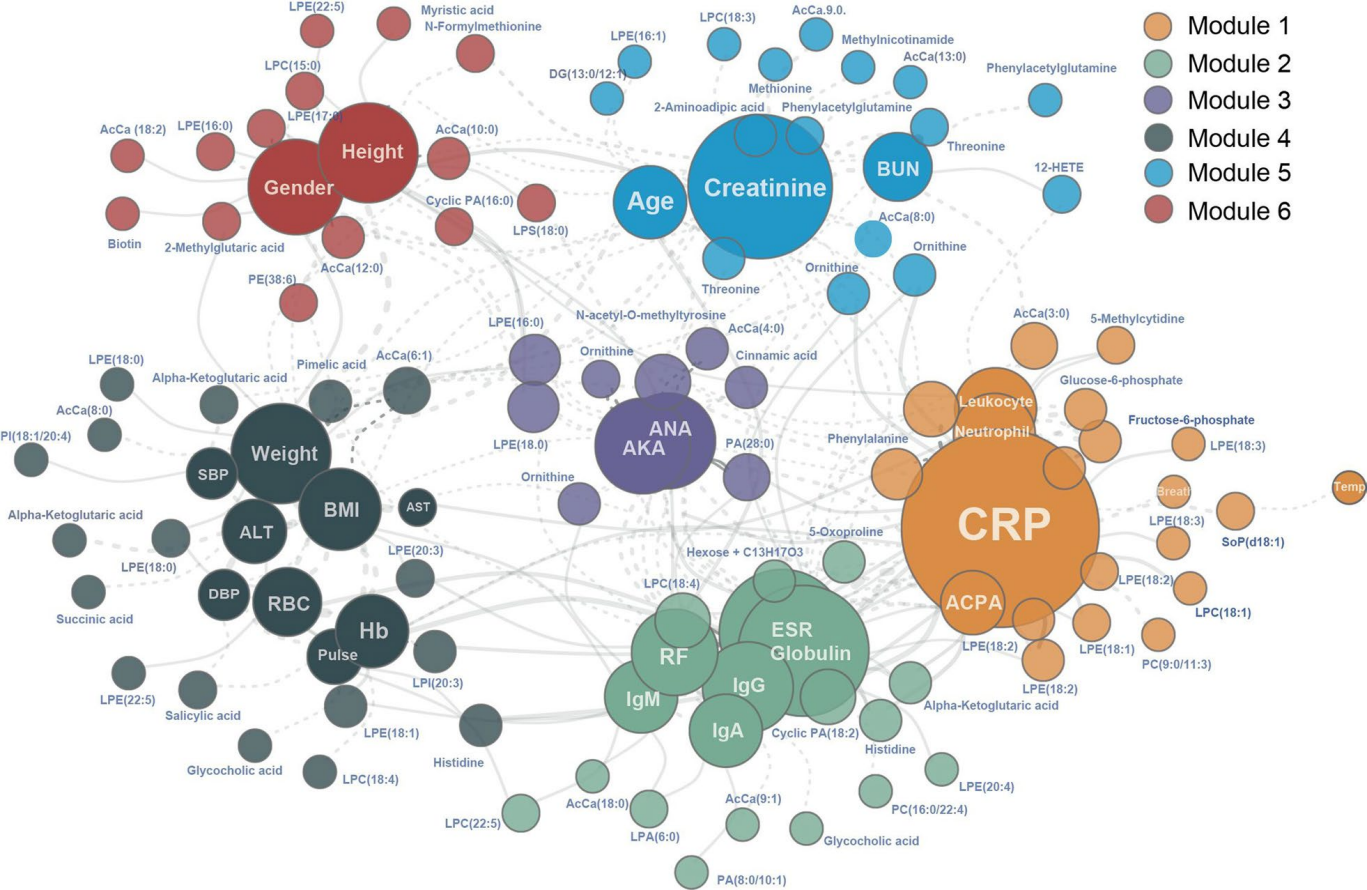
A co-occurrence network showing correlation between clinical parameters and specific serum metabolites and lipids. Nodes represent clinical parameters or metabolites and lipids, and two nodes are connected if they are significantly correlated (adjusted p-value < 0.05, r-value > 0.2). The solid line signifies a positive correlation, and the dotted line signifies a negative correlation. The size of each node is proportional to the number of connections (that is, degree). Nodes colored by modules

To understand the association of MLP with clinical parameters of RA, we used a correlation-based network approach to explore the co-occurrence patterns between 28 clinical variables and serum concentrations of 265 identified metabolites and lipids in the RA patients, which could help to explore the correlation of metabolic products and clinical confounders. In the co-occurrence network (Fig. 3), nodes represent clinical parameters or metabolites and lipids, and two nodes are connected if they are significantly correlated (adjusted p-value < 0.05 and r-value > 0.2). The resulting network consisted of 117 nodes and 274 edges. The modularity index was 0.467, which is above 0.4 suggested for a modularly structured network [24]. Overall, the entire network could be parsed into six modules. As shown in Fig. 3, module 1 contained CRP, leukocyte, neutrophil and ACPA related to inflammatory activity and 17 metabolites and lipids. Modules 2 and 3 contained RF, IgG, IgM, IgA, globulin, ESR, ANA and AKA related to the immune response and 22 metabolites and lipids. The general clinical variables such as age, gender and BMI were clustered in modules 4–5 and were far away from modules 1–3. Therefore, the co-occurrence network analysis revealed that there was a clear association between the serum inflammation/immune markers and concentrations of metabolites and lipids in the RA patients.

### Stratification of disease activity using MLP

**Fig. 4:**
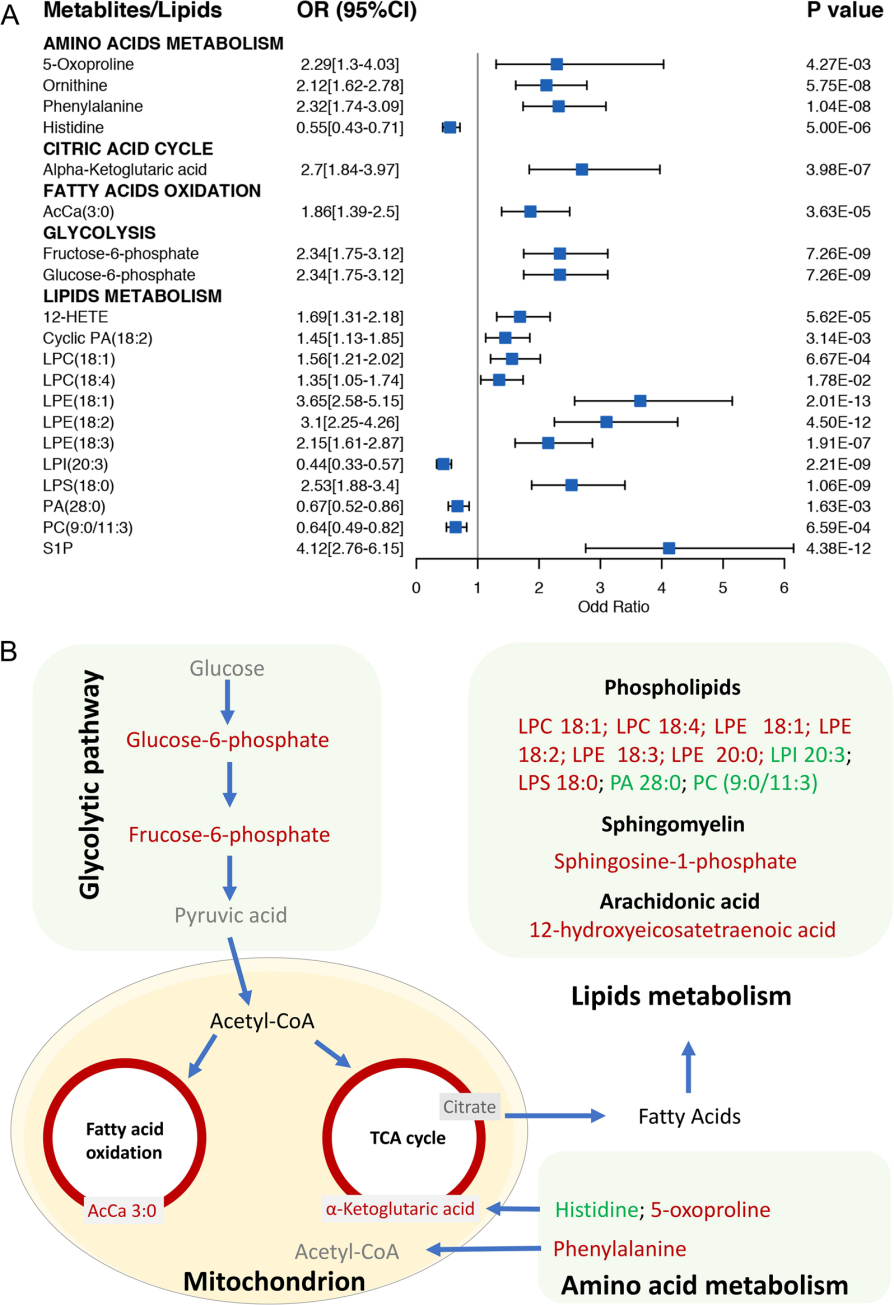
Association between the inflammation-immune activity and aberrant metabolism. **A** Serum metabolites and lipids levels were associated with the risk for RA according to relative peak intensity from untargeted mass spectrometry analyses of subjects (N = 325). **B** RA-associated metabolite and lipids in cellular metabolic pathways. Upregulated metabolites or lipids were colored in red and downregulated metabolites or lipids were colored in green

**Fig. 5:**
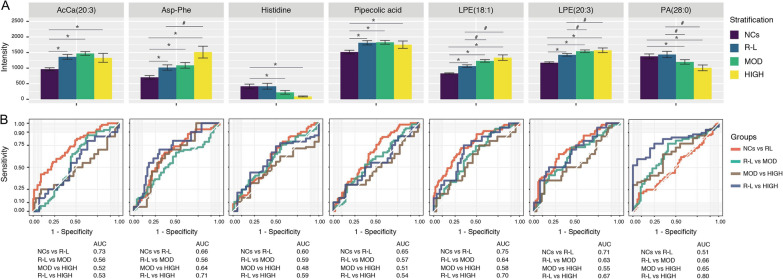
Metabolites and lipids correlated with RA disease activity. Boxplots (**A**) and ROC analysis (**B**) of seven metabolites and lipids with differential levels among normal control group (NCs) and RA with low disease activity (R-L), moderate disease activity (MOD) and high disease activity (HIGH). # p < 0.05 versus NCs group, *p < 0.05 versus R-L group. Data are presented as mean ± SEM, and analyzed by Wilcoxon−Mann U test with FDR control

To analyze the utility of using MLP for stratification of RA cases based on disease activity, twenty metabolites and lipids strongly correlated to the inflammatory/immune activity were selected from modules 1 and 2 in the co-occurrence network (Fig. 3). Sixteen metabolites and lipids had significantly increased odd ratios for RA status, while 4 metabolites and lipids had significantly decreased odd ratios (Fig. 4). Disease activity score DAS28-CRP is often utilized to evaluate the disease activity of RA patients [25, 26]. Using DAS28-CRP to stratify RA patients, we further identify 7 metabolites and lipids that were strongly associated with disease activity categories by using the ordinal regression method (Fig. 5). Among these, AcCa (20:3), aspartyl-phenylalanine (asp-phe), pipecolic acid, lysophosphatidylethanolamine (LPE 18:1) and LPE (20:3) appeared to be positively correlated with higher RA disease activity, while histidine and PA (28:0) were negatively correlated with RA disease activity (Fig. 5A). AcCa (20:3), LPE (18:1) and LPE (20:3) were significantly increased in the remission-low risk (R-L) group compared to the NCs group (p < 0.05, AUC > 0.7). A small peptide asp-phe and lipid LPE (18:1) were significantly increased in the DAS28-CRP high disease activity (HIGH) group compared with the R-L group (p < 0.05, AUC > 0.7), indicating their potential role as biomarkers in RA disease activity stratification (Fig. 5B).

## Discussion

RA is a highly heterogeneous disease with variable presenting symptoms, severity and response to treatment. Autoimmune damage may happen years before symptoms occur and clinical diagnosis is made. It is important for early and accurate diagnosis of RA and prompt initiation of effective treatment to prevent joint damage and functional loss [27]. Current diagnostic criteria are based on comprehensive evaluation of symptoms, serology status and acute phase reactant levels. Because a significant percentage of RA patients are negative for serologic markers, additional diagnostic methods are actively being developed to help increase diagnostic accuracy, particularly for seronegative RA patients. The chronic inflammation and joint destruction in RA patients may cause metabolic perturbations in the peripheral blood, providing opportunities to discover potential biomarkers to improve the clinical diagnosis of RA [4, 28].

Previous studies have reported metabolic changes in RA by using nuclear magnetic resonance (NMR) or RPLC-MS [4, 10, 11]. We developed a multi-platform strategy including two reverse phase and two HILIC chromatography hyphenated to high-resolution mass spectrometry (HRMS) in positive and negative ionization modes for the simultaneous quantitative analysis of metabolites and lipids. By means of the integrative metabolomic and lipidomic analysis, we were able to identify 26 serum metabolites and lipids that correctly classified RA patients from normal control subjects. A multivariate classification model was derived from the discovery set and was subsequently validated using an independent validation set. It is noteworthy that our model combined with RF test reached 100% validation accuracy, and all patients with seronegative RA were identified by using our model, thus showing the potential for providing a diagnostic tool in both seropositive and seronegative RA.

Similar findings pertaining to the differences in the levels of lysine, phenylalanine, proline, ornithine and salicylic acid between the RA patients and normal control subjects were observed previously [11, 29]. These metabolites have been associated with immune system activation [4, 30, 31]. On the other hand, we found serum level of histidine, which has anti‐inflammatory effects and antioxidative stress effect via inhibiting peroxisome proliferator-activated receptor γ (PPARγ) -involved pathways [32], was significantly decreased in RA patients. The role of histidine in RA needs further studies since histidine supplementation did not show an advantage in a double-blind trial [33]. In our study histidine level was inversely correlated with the RA disease activity defined by DAS28-CRP. Histidine level was significantly decreased in patients with moderate and high disease activity, while no significant changes were seen in RA patients with remission and low disease activity (Fig. 5A).

Lipids are implicated in diverse biological functions, including being the major component of cell membranes and the regulation of cell migration and inflammation [7, 34]. Our study found that increased levels of lipid mediators such as sphingosine 1-phosphate (S1P) and 12-HETE and lysophospholipids such as LPE (18:1), LPE (18:2), lysophosphatidylcholine (LPC 18:1), LPC (18:4) and lysophosphatidylserine (LPS 18:0) in RA patients were positively correlated with CRP levels (Fig. 3), supporting their roles in inflammation. Other groups have reported that LPC was shown to induce cyclooxygenase-2 expression in the vascular endothelial cells, playing a possible pro-inflammatory role [35]. S1P is generated from sphingosine by activation of sphingosine kinase and has been implicated as a potential therapeutic target in RA [36]. Moreira et al. reported that 12-HETE, an arachidonic acid-derived metabolite, may play an important role in the chronic inflammatory process associated with RA by mediating proinflammatory actions [37].

Ongoing inflammation and immune activation are characterized by rising energy demand that is required for immune cell growth, proliferation and the production of proinflammatory molecules [38]. In our study, the increased levels of metabolites (e.g., alpha-ketoglutaric acid, AcCa (3:0), fructose-6-phosphate and glucose-6-phosphate) seen in the serum of RA patients indicate enhanced energy metabolism involving glycolysis, citric acid cycle and fatty acid oxidation (Fig. 4). Methotrexate (MTX), a first line treatment for RA, is an antimetabolite drug that targets folic acid metabolism. Many ongoing clinical trials are investigating potential drugs that target lipid/glucose metabolic pathways to improve inflammation control and disease outcome [5]. In addition, using metabolomic approach to delineate inflammation-associated metabolic status of RA patients may offer a promising method for disease activity and treatment effect monitoring.

The strengths of our study include that it employs a comprehensive multi-platform approach for metabolomic and lipidomic analysis, large sample size and its potential to provide diagnostic value for both seropositive and seronegative RA patients. Its limitations include that it is still a discovery study that shows correlation of serum markers to RA, but does not directly address the mechanisms of these markers in RA. The diagnostic value of the discovered metabolomic and lipidomic markers for discriminating between seronegative RA and other types of inflammatory arthritis such as psoriatic arthritis, reactive arthritis or spondyloarthritis will be investigated in future studies. For developing our method into clinical practice, our classification model still needs to be evaluated in other multinational/multiethnic cohorts.

In conclusion, we have built a serum metabolic/lipidomic-markers-based model that has potential diagnose value. Our study suggests the integrative comprehensive metabolomic and lipidomic profiling is a promising system biology approach for uncovering biomarkers useful for RA diagnosis and disease activity monitoring.

## Supplementary Information

**Additional file 1.** Materials and Methods, Quality Control, Tables S1–S3, Figure S1–S3.

## Data Availability

All data generated or analyzed during this study are included in this published article [and its Additional file].
