# Supplementary material for: Serum metabolomic and lipidomic profiling identifies diagnostic biomarkers for seropositive and seronegative rheumatoid arthritis patients
Source: J Transl Med. 2021 Dec 7;19:500. doi: 10.1186/s12967-021-03169-7 (PMC8650414; doi:10.1186/s12967-021-03169-7)
Supplement: Supplementary file 1 — Additional file 1. Materials and Methods, Quality Control, Tables S1–S3, Figure S1–S3. [file 12967_2021_3169_MOESM1_ESM.pdf]

1. LC-MS analysis

Venous blood was collected in the morning before breakfast from all the participants, and then serum samples were separated at 2200*g* for 5 min at 4°C and stored at −80°C until use. For the polar metabolite profiling, dried samples were reconstituted in 100μl of 80% methanol and analyzed by using a Dionex U3000 LC system coupled online to a Q Exactive Orbitrap MS instrument (Thermo Fisher Scientific, MA, USA) set at 35000 resolution (at m/z 200). The mass scanning range was 70–1000 m/z and the capillary temperature was 350°C. Nitrogen sheath gas was set at a flow rate of 30 L/min. Nitrogen auxiliary gas was set at a flow rate of 10 L/min. Spray voltage was set to 4.5 kV and 3.0 kV for positive or negative ion mode, respectively. Solvent A was: water with 10 mM ammonium carbonate and 0.2% ammonium hydroxide for method 1 with basic conditions; water with 10 mM ammonium formate and 0.1% formic acid (v/v) for method 2 with acidic conditions; Solvent B was: acetonitrile for method 1 with basic conditions and acetonitrile with 0.1% formic acid (v/v) for method 2 with acidic conditions. An Waters BEH amide (50 × 2.1 mm, 1.7 μm) was used for analysis. The flow rate was 0.2 mL/min. For the lipids profiling, dried samples were reconstituted in 100μl of acetonitrile for instrument analysis. The MS instrument was carried out as described above. LC solvents A consisted of ACN/H2O (6:4, v/v) with 0.1% formic acid. LC solvents B consisted of isopropanol/ACN (9:1, v/v) with 10mM ammonium formate. Method 3 and method 4 were performed with the same solvents system. A Waters BEH C18 (50 × 2.1 mm, 1.7 μm) was used for analysis. The flow rate was 0.2 mL/min.

2. Quality control approach

To obtain high quality data comparable to the metabolomic and lipidomic profiling, a quality assessment strategy based on the periodic analysis of quality control (QC) samples together with serum samples was performed as our previous study (Luan, et al., 2015). The QC samples consisted of mixing equal volumes of serum obtained from RA patients and normal control subjects before sample preparation as they were aliquoted for analysis. This pooled QC sample was prepared as described for real samples and used to estimate a "mean" profile representing all

the peaks detected during the MS analysis. At the beginning of run, five QC samples were advisable to equilibrate the analytical platform and then injected at regular intervals (e.g., every eight real samples) throughout the analytical run in order to provide data. Quality assurance was achieved by the statTarget package as follows (Luan, et al., 2018). Briefly, peaks with more than 50% missing values were removed. The intensity of remaining peaks in samples was corrected according to the QC-RFSC algorithm. Only peaks in QC samples with a relative standard deviation of less than 30% were further used for statistical analysis. The repeatability of data can be assessed and the intra-variation also can be corrected using QC-RFSC algorithm. Principle component analysis (PCA) was performed on UV-scaled data to visualize general clustering of QC samples together with all samples on the scores plot. PCA score plot representation of QC samples showed no drift during the LC-MS analysis as shown in Fig. S3. Thus, reproducibility and stability of metabolic features were acceptable and subsequently used for statistical analysis.

Supplemental Tables

Table S1. The identified 265 individual metabolites and lipids in the peak table

ID	VIP	FoldChanges	Adjusted.pvalue	Name	Class
X183	1.189	0.553	0.001	Acetylcarnitine	acyl carnitines
X191	2.124	0.578	0.000	O-propanoyl-carnitine	acyl carnitines
X196	1.745	0.613	0.000	O-butanoyl-carnitine	acyl carnitines
X202	0.973	0.666	0.016	2-Methylbutyroylcarnitine	acyl carnitines
X206	2.429	2.067	0.000	O-octanoyl-R-carnitine	acyl carnitines
X210	2.828	2.273	0.000	Dimethylheptanoyl carnitine	acyl carnitines
X211	0.709	0.915	0.469	Decadienoylcarnitine	acyl carnitines
X212	1.251	2.127	0.000	O-decanoyl-R-carnitine	acyl carnitines
X213	0.170	0.927	0.861	Dimethylnonanoyl carnitine	acyl carnitines
X218	2.408	1.859	0.000	2-Hydroxylauroylcarnitine	acyl carnitines
X227	1.502	1.452	0.000	(3R)-3-Tridecanoyloxy-4-(trimethylazaniumyl)butanoate	acyl carnitines
X236	1.966	0.709	0.000	Octadecadienoyl carnitine	acyl carnitines
X239	1.771	0.705	0.000	Elaidic carnitine	acyl carnitines
X242	1.612	0.781	0.000	Stearoylcarnitine	acyl carnitines
X257	2.275	0.611	0.000	Eicosatrienoylcarnitine	acyl carnitines
X266	0.455	0.925	0.431	Arachidyl carnitine	acyl carnitines
X306	0.069	1.125	0.717	Behenoylcarnitine	acyl carnitines
X33	0.321	0.936	0.072	Carnitine	acyl carnitines
X355	0.459	0.894	0.057	C24:1-Carnitine	acyl carnitines
X457	0.908	1.175	0.007	tetradecadienoylcarnitine	acyl carnitines
X489	0.696	1.039	0.533	tetradecanoylcarnitine	acyl carnitines
X60	2.033	0.568	0.000	Hexenoylcarnitine	acyl carnitines
X61	0.244	1.072	0.661	Hexanoylcarnitine	acyl carnitines
X64	0.981	0.763	0.008	octenoylcarnitine	acyl carnitines
X67	1.660	1.533	0.000	Nonenoylcarnitine	acyl carnitines
X709	0.414	0.963	0.960	Palmitoylcarnitine	acyl carnitines
X15	0.021	1.021	0.920	Nicotinamide	Amines
X189	0.256	0.933	0.029	Kynurenine	Amines
X190	0.574	0.912	0.041	Nicotinoylcholine	Amines
X199	1.397	0.597	0.000	Biotin	Amines
X20	1.046	1.376	0.000	Methylnicotinamide	Amines
X275	0.594	0.909	0.369	4-Methylcatechol	Amines
X46	1.102	1.805	0.005	Cotinine	Amines
X637	0.948	0.732	0.001	Acetylcholine	Amines
X689	0.022	0.985	0.920	Creatinine	Amines
X8	1.468	0.469	0.002	Choline	Amines
X12	2.020	0.670	0.000	Threonine	Amino acids
X16	1.063	0.707	0.000	5-Oxoproline	Amino acids
X168	0.555	1.072	0.204	Acetylleucine	Amino acids
X18	0.877	0.833	0.000	Aspartic acid	Amino acids
X181	0.245	2.147	0.920	N,N-Dimethylarginine	Amino acids
X194	0.528	0.903	0.080	Leucylproline	Amino acids
X197	0.728	0.739	0.123	Cystine	Amino acids
X2	1.073	1.279	0.008	Alanine	Amino acids
X204	0.840	1.069	0.161	Glutamylleucine	Amino acids

X205	1.908	0.548	0.000	Asp-Phe	Amino acids
X24	1.113	0.736	0.000	Glutamine	Amino acids
X27	1.666	0.751	0.000	Methionine	Amino acids
X315	1.117	2.357	0.000	Histidine	Amino acids
X32	0.357	0.922	0.298	Acetylproline	Amino acids
X324	0.214	1.079	0.910	Isovalerylglycine	Amino acids
X375	1.125	0.744	0.000	N-Formylmethionine	Amino acids
X44	0.881	0.734	0.246	Citrulline	Amino acids
X516	0.953	0.703	0.044	Taurine	Amino acids
X613	2.348	0.593	0.000	Ornithine	Amino acids
X62	2.448	0.326	0.000	Phenylalanylisoleucine	Amino acids
X625	0.490	0.920	0.328	Aminobenzoic acid	Amino acids
X650	1.091	1.230	0.006	Serine	Amino acids
X681	1.846	0.700	0.000	N-acetyl-O-methyltyrosine	Amino acids
X682	2.221	0.450	0.000	Phenylacetylglutamine	Amino acids
X711	1.281	0.536	0.001	Acetylglycine	Amino acids
X732	1.970	0.781	0.000	Phenylalanine	Amino acids
X739	1.182	0.707	0.000	Lysine	Amino acids
X756	0.213	0.780	0.651	Acetylcysteine	Amino acids
X757	0.618	0.959	0.035	Arginine	Amino acids
X758	0.685	0.907	0.044	Leucine/Isoleucine	Amino acids
X763	1.666	1.362	0.000	Valine	Amino acids
X765	0.825	0.904	0.000	Glycine	Amino acids
X766	0.826	0.940	0.026	Glutamic acid	Amino acids
X770	0.667	0.918	0.006	Tyrosine	Amino acids
X771	0.685	1.093	0.181	Tryptophan	Amino acids
X772	0.763	0.900	0.000	Asparagine	Amino acids
X776	1.691	0.744	0.000	Proline	Amino acids
X108	0.582	0.601	0.002	Deoxycholic Acid	bile acids
X110	0.826	1.003	0.267	Dehydrocholic acid	bile acids
X118	1.261	1.319	0.001	Glycocholic acid	bile acids
X333	0.463	1.752	0.730	Glycolic acid	Bile acids
X103	1.525	0.739	0.000	12-hydroxyeicosatetraenoic acid	Fatty acyls
X274	1.016	0.378	0.005	DG(13:0/12:1)	glycerolipids
X74	1.832	1.482	0.001	DAG(18:1)	glycerolipids
X112	0.551	0.611	0.008	LPA(16:0)	glycerophospholipids
X119	0.080	1.512	0.893	LPA(20:0)	glycerophospholipids
X120	0.210	1.087	0.753	PE(17:0)	glycerophospholipids
X122	0.830	1.130	0.391	PS(16:0)	glycerophospholipids
X123	1.361	1.145	0.047	PA(23:5)	glycerophospholipids
X132	0.776	1.191	0.659	LPI(16:0)	glycerophospholipids
X133	1.018	1.377	0.001	PA(28:0)	glycerophospholipids
X134	0.396	1.081	0.945	LPI(18:2)	glycerophospholipids
X135	1.358	1.298	0.000	PC(20:4)	glycerophospholipids
X136	0.691	1.155	0.957	PC(20:3)	glycerophospholipids
X138	0.313	1.270	0.086	PE(38:7)	glycerophospholipids
X140	1.621	0.531	0.000	PE(38:6)	glycerophospholipids
X141	0.986	1.135	0.001	PE(38:5)	glycerophospholipids
X142	1.753	0.687	0.000	PE(18:1)	glycerophospholipids

X143	1.643	0.792	0.000	PC(34:7)	glycerophospholipids
X145	0.531	0.886	0.030	PC(34:6)	glycerophospholipids
X156	1.128	0.869	0.007	PI(36:4)	glycerophospholipids
X234	1.579	0.763	0.000	PA(8:0/10:1)	glycerophospholipids
X240	1.300	0.864	0.000	PA(8:0/10:0)	glycerophospholipids
X243	2.390	0.722	0.000	LPE(16:0)	glycerophospholipids
X255	1.442	0.493	0.000	PA(8:0/12:4)	glycerophospholipids
X260	1.967	0.459	0.000	LPE(16:1)	glycerophospholipids
X278	2.035	0.579	0.000	LPE(18:3)	glycerophospholipids
X284	2.443	0.641	0.000	LPE(18:2)	glycerophospholipids
X294	0.223	3.855	0.824	PA(8:0/12:0)	glycerophospholipids
X297	1.931	0.619	0.000	LPE(17:0)	glycerophospholipids
X300	2.293	0.656	0.000	PA(6:0/14:3)	glycerophospholipids
X302	1.522	0.629	0.000	LPC(15:0)	glycerophospholipids
X311	2.665	0.676	0.000	LPE(18:1)	glycerophospholipids
X313	0.680	0.967	0.030	LPC(14:0)	glycerophospholipids
X316	1.156	0.866	0.021	LPC(16:1)	glycerophospholipids
X317	0.386	0.979	0.717	PA(4:0/16:0)	glycerophospholipids
X319	0.888	1.004	0.932	LPC(16:0)	glycerophospholipids
X320	1.952	0.791	0.000	LPE(18:0)	glycerophospholipids
X328	0.795	0.775	0.044	LPA(22:6)	glycerophospholipids
X335	1.229	2.185	0.000	LPC(18:2)	glycerophospholipids
X338	0.804	1.099	0.003	PC(8:0/9:0)	glycerophospholipids
X341	2.324	0.789	0.000	LPE(20:4)	glycerophospholipids
X344	2.557	0.614	0.000	LPE(20:3)	glycerophospholipids
X349	1.150	1.232	0.001	LPC(18:0)	glycerophospholipids
X353	0.729	0.961	0.252	LPE(20:1)	glycerophospholipids
X356	0.602	0.982	0.165	LPG(18:2)	glycerophospholipids
X358	1.441	0.794	0.000	LPC(18:4)	glycerophospholipids
X362	1.500	0.838	0.048	LPG(18:0)	glycerophospholipids
X372	1.094	0.588	0.004	LPC(18:3)	glycerophospholipids
X381	2.108	0.679	0.000	LPS(18:0)	glycerophospholipids
X387	0.691	1.104	0.486	PA(4:0/20:5)	glycerophospholipids
X394	1.412	0.707	0.000	LPE(22:6)	glycerophospholipids
X397	2.187	0.706	0.000	LPE(22:5)	glycerophospholipids
X400	1.179	1.374	0.004	LPC(17:0)	glycerophospholipids
X406	0.887	1.054	0.273	LPC(19:1)	glycerophospholipids
X410	1.293	0.944	0.772	LPC(17:1)	glycerophospholipids
X421	0.179	0.974	0.853	LPC(20:3)	glycerophospholipids
X432	0.493	1.124	0.735	LPC(20:1)	glycerophospholipids
X433	1.363	0.758	0.000	LPC(18:1)	glycerophospholipids
X438	1.338	1.130	0.013	LPC(20:0)	glycerophospholipids
X441	1.191	1.479	0.001	LPC(19:0)	glycerophospholipids
X444	0.280	1.033	0.932	LPC(21:0)	glycerophospholipids
X450	1.540	1.216	0.000	LPC(22:5)	glycerophospholipids
X453	0.490	0.914	0.002	LPC(22:4)	glycerophospholipids
X462	1.029	0.847	0.001	LPC(22:2)	glycerophospholipids
X465	0.799	1.665	0.588	LPC(22:1)	glycerophospholipids
X472	0.834	0.782	0.022	LPI(16:1)	glycerophospholipids

X477	0.509	1.066	0.144	LPC(22:6)	glycerophospholipids
X478	0.410	1.062	0.204	LPC(20:5)	glycerophospholipids
X490	0.383	0.968	0.526	LPC(32:1)	glycerophospholipids
X491	1.178	0.731	0.000	Cyclic PA(16:0)	glycerophospholipids
X495	1.704	0.834	0.000	LPC(20:2)	glycerophospholipids
X503	0.692	1.147	0.053	LPI(17:0)	glycerophospholipids
X505	0.518	1.036	0.422	LPC(20:4)	glycerophospholipids
X506	0.205	1.029	0.834	PE(18:1/18:2)	glycerophospholipids
X509	0.719	0.795	0.163	LPI(18:3)	glycerophospholipids
X513	0.165	0.997	0.966	LPI(18:1)	glycerophospholipids
X515	0.586	1.037	0.330	LPC(34:1)	glycerophospholipids
X517	1.068	1.223	0.013	LPI(18:0)	glycerophospholipids
X518	0.434	1.535	0.474	PE(16:0/22:6)	glycerophospholipids
X519	1.135	1.206	0.003	PC(9:0/11:3)	glycerophospholipids
X522	0.267	0.966	0.700	PC(16:0/18:1)	glycerophospholipids
X525	1.074	1.223	0.010	PA(20:5/11:4)	glycerophospholipids
X526	1.398	1.182	0.000	PC(18:1/18:0)	glycerophospholipids
X528	0.402	1.028	0.451	PE(18:0/22:6)	glycerophospholipids
X529	0.928	1.112	0.062	LPI(20:4)	glycerophospholipids
X530	0.388	0.841	0.557	PC(16:0/18:2)	glycerophospholipids
X533	2.118	1.383	0.000	LPI(20:3)	glycerophospholipids
X535	0.352	1.033	0.625	LPI(22:6)	glycerophospholipids
X546	0.407	1.118	0.548	PE(16:0/18:2)	glycerophospholipids
X547	0.422	1.042	0.643	PC(16:0/20:3)	glycerophospholipids
X551	0.637	1.058	0.411	PC(18:0/18:1)	glycerophospholipids
X554	1.077	1.465	0.004	PE(16:0/20:4)	glycerophospholipids
X558	0.305	0.806	0.671	PC(18:1/21:0)	glycerophospholipids
X560	0.171	0.780	0.930	PC(18:2/20:4)	glycerophospholipids
X561	0.721	1.129	0.108	PE(18:1/18:2)	glycerophospholipids
X564	0.139	0.980	0.736	PC(16:0/22:5)	glycerophospholipids
X566	0.725	1.207	0.051	PC(18:0/20:3)	glycerophospholipids
X567	0.479	1.290	0.343	PE(18:0/20:4)	glycerophospholipids
X572	1.004	1.274	0.009	PC(29:1/11:3)	glycerophospholipids
X573	0.499	1.146	0.137	PE(18:1/20:4)	glycerophospholipids
X577	0.136	0.938	0.981	ST(18:2/22:0)	glycerophospholipids
X578	1.222	0.266	0.001	PA(8:0/8:0)	glycerophospholipids
X598	1.142	1.499	0.008	PA(4:0/11:0)	glycerophospholipids
X610	1.227	1.712	0.001	PC(16:0/18:1)	glycerophospholipids
X612	0.893	0.847	0.004	PC(18:2/18:2)	glycerophospholipids
X616	1.043	1.188	0.002	PC(18:0/18:2)	glycerophospholipids
X619	0.534	0.889	0.238	PA(8:0/8:0)	glycerophospholipids
X622	1.250	0.701	0.000	PI(16:0/18:2)	glycerophospholipids
X626	1.469	1.226	0.000	PC(16:0/22:4)	glycerophospholipids
X632	0.738	0.756	0.184	PI(16:0/20:4)	glycerophospholipids
X633	0.537	0.833	0.026	PI(16:0/20:3)	glycerophospholipids
X636	1.344	1.488	0.000	PC(18:0/22:6)	glycerophospholipids
X640	1.531	0.793	0.000	PI(18:1/20:4)	glycerophospholipids
X641	0.566	0.837	0.069	PI(16:1/22:3)	glycerophospholipids
X642	0.641	0.746	0.290	PI(18:1/20:3)	glycerophospholipids

X669	1.246	0.776	0.009	Cyclic PA(18:2)	glycerophospholipids
X673	0.626	0.681	0.184	cPA(18:2)	glycerophospholipids
X687	0.842	1.516	0.054	cPA(18:0)	glycerophospholipids
X688	1.405	1.293	0.001	LPA(6:0)	glycerophospholipids
X707	1.110	1.197	0.000	LPC(9:0)	glycerophospholipids
X96	0.822	1.210	0.009	PE(36:5)	glycerophospholipids
X215	0.733	1.106	0.162	WE(2:0/18:1)	Lipids
X220	1.141	0.913	0.001	WE(2:0/20:3)	Lipids
X222	0.610	1.352	0.050	WE(2:0/20:2)	Lipids
X224	0.536	0.763	0.079	WE(2:0/20:1)	Lipids
X474	0.718	0.878	0.083	BisMePA(4:0/20:0)	Lipids
X480	0.312	0.937	0.167	BisMePA(4:0/21:1)	Lipids
X483	1.583	0.844	0.000	BisMePA(4:0/21:0)	Lipids
X485	2.125	0.724	0.000	BisMePA(4:0/22:3)	Lipids
X720	0.819	0.736	0.040	WE(4:0/23:2)	Lipids
X203	1.157	0.792	0.001	5-Methylcytidine	nucleotides
X464	0.982	0.615	0.000	Uridine	nucleotides
X623	0.800	0.743	0.010	Hypoxanthine	nucleotides
X684	0.355	0.970	0.903	Inosine	nucleotides
X99	0.897	0.104	0.000	Cytidine-3',5'-cyclicmonophosphate	nucleotides
X167	1.055	0.922	0.006	Homogentisic acid	Organic acids
X17	0.902	0.804	0.011	Pyroglutamic acid	Organic acids
X174	0.323	0.934	0.936	Sarcosine	Organic acids
X21	0.338	0.917	0.799	Urocanate	Organic acids
X235	0.755	1.034	0.000	Nicotinic acid	Organic acids
X248	1.804	0.703	0.000	Pipecolic acid	Organic acids
X276	0.612	0.843	0.277	2-Hydroxy-4-methylpentanoic acid	Organic acids
X277	0.192	2.375	0.800	4-Hydroxybenzoic acid	Organic acids
X280	0.372	0.902	0.584	4-Acetamidobutyric acid	Organic acids
X305	1.670	2.343	0.002	Hydroxyphenylacetic acid	Organic acids
X329	0.878	0.982	0.992	3-Methyladipic acid	Organic acids
X331	0.550	0.970	0.172	3-Hydroxy-3-methylglutaric acid	Organic acids
X373	0.848	0.428	0.015	Aconitic acid	Organic acids
X379	1.130	0.393	0.001	Hippuric acid	Organic acids
X405	0.130	0.490	0.659	Indole-3-propionic acid	Organic acids
X411	0.935	0.622	0.009	Citric acid	Organic acids
X446	1.498	1.284	0.000	Myristic acid	Organic acids
X514	0.214	0.716	0.005	Lactic acid	Organic acids
X542	1.123	0.401	0.024	2-Methylglutaric acid	organic acids
X587	1.557	0.595	0.000	Cinnamic acid	organic acids
X595	0.966	0.520	0.001	2-Hydroxyisobutyric acid	organic acids
X629	1.065	1.436	0.028	Salicylic acid	organic acids
X652	1.173	1.283	0.017	Pimelic acid	Organic acids
X655	1.659	0.729	0.000	2-Aminoadipic acid	Organic acids
X657	0.948	0.741	0.009	2-Hydroxycinnamic acid	Organic acids
X660	0.388	1.760	0.045	Aminobutyric acid	Organic acids
X666	0.636	0.850	0.070	Pyridoxic acid	Organic acids
X674	1.149	0.889	0.111	Indolelactic acid	Organic acids
X676	0.190	0.897	0.296	3-Hydroxysebacic acid	Organic acids

X693	1.114	1.029	0.356	Ketoisovaleric acid	Organic acids
X695	2.129	0.277	0.000	Acetylneuraminic acid	Organic acids
X696	1.634	0.474	0.000	Mycophenolic acid	Organic acids
X698	1.046	0.794	0.026	Succinic acid	Organic acids
X701	1.346	0.907	0.000	5-Aminovaleric acid	Organic acids
X717	0.382	0.905	0.738	Hydroxyvaleric acid	Organic acids
X742	1.888	0.685	0.000	Alpha-Ketoglutaric acid	Organic acids
X744	0.779	0.587	0.000	Fumaric acid	Organic acids
X745	0.816	0.752	0.000	Malic acid	Organic acids
X157	0.457	0.852	0.179	MGDG(38:6)	saccharolipids
X552	0.274	1.036	0.509	MGMG(28:0)	saccharolipids
X90	2.451	0.689	0.000	MGDG(22:2)	saccharolipids
X95	1.005	1.275	0.019	MGDG(25:6)	saccharolipids
X208	1.184	0.575	0.001	So(d16:0)	sphingolipids
X209	0.905	0.927	0.014	So(d18:1)	sphingolipids
X426	2.340	0.632	0.000	SoP(d18:1)	sphingolipids
X510	0.347	1.042	0.356	SM(18:1/18:1)	sphingolipids
X537	0.476	0.759	0.720	SoP(d20:0)	sphingolipids
X550	0.582	0.982	0.116	SM(13:0/18:1)	sphingolipids
X581	0.668	0.893	0.064	SM(18:0/18:2)	sphingolipids
X628	0.377	0.974	0.864	SM(22:2/20:1)	sphingolipids
X631	0.570	3.419	0.072	SM(24:0/18:2)	sphingolipids
X109	2.255	2.218	0.000	Hexose + C13H17O3	sugar derivatives
X392	0.735	1.191	0.005	Mannitol	sugar derivatives
X48	1.109	1.619	0.204	Galactosamine	sugar derivatives
X747	2.017	0.584	0.000	Glucose-6-phosphate	sugar derivatives
X748	2.017	0.584	0.000	Fructose-6-phosphate	sugar derivatives
X751	0.342	0.896	0.469	Glucose	sugar derivatives

*SM, Sphingomyelin; So, sphingosine; SoP, Sphingosine phosphate; MGDG, Monogalactosyldiacylglycerol; BisMePA, bismethyl phosphatidic acid; cPA, cyclic phosphatidic acid; PC, phosphatidylcholine; PE, phosphatidylethanolamine; PG, phosphatidylglycerol; PI, phosphatidylinositol; LPC, lysophosphatidylcholine; LPS, lysophosphatidylserine; LPI, lysophosphatidylinositol; We, wax ester; PA, phosphatidic acid.

Table S2. Classification table of the validation cohort

Clinical diagnosis	Prediction		Total
	RA	NCs	
RA	48	5	53
NCs	3	26	29

Table S3. Analysis of the misclassificated cases

Sample ID	Disease Status	Gender	Misclassified prediction responses	RF (IU/ml)	ACPA (IU/ml)	RF-adjusted results
S174	RA	Female	Negative	Positive (21.1)	Negative (0)	aRA
S254	RA	Male	Negative	Positive (1920)	Positive (200)	aRA
S270	RA	Female	Negative	Positive (134)	Positive (200)	aRA
S273	RA	Female	Negative	Positive (20)	Negative (0.5)	aRA
S285	RA	Female	Negative	Positive (20)	Negative (0.5)	aRA
S3	NCs	Male	Positive	-	-	-
S322	NCs	Female	Positive	-	-	-
S83	NCs	Male	Positive	-	-	-

Rheumatoid factor (RF) values ≥ 20 are considered positive, and ACPA values ≥ 5 are considered positive. aRA denotes RF-adjusted classification status.

Supplemental Figures

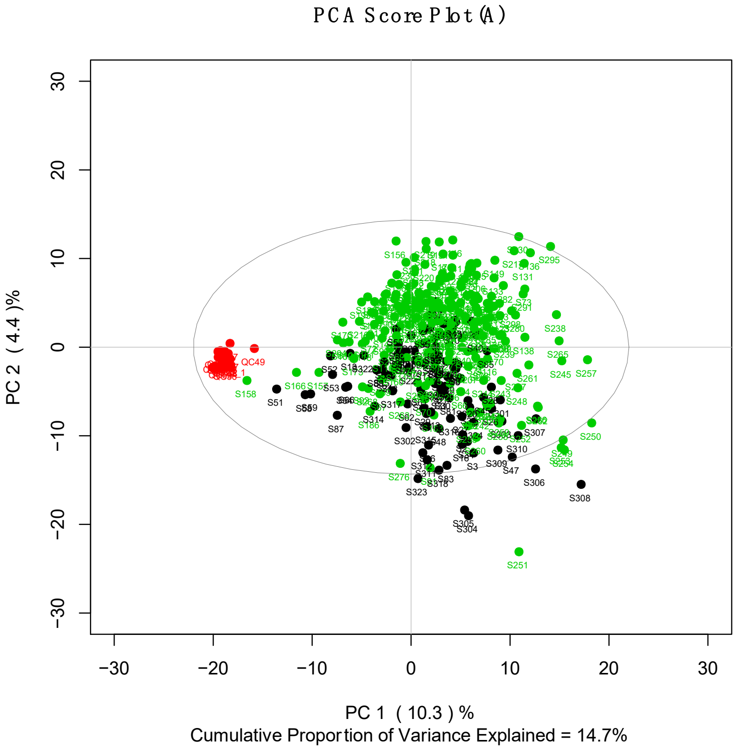

Fig. S1. Score plot of PCA analysis showing the good data quality of this study. Red color denotes QC samples.

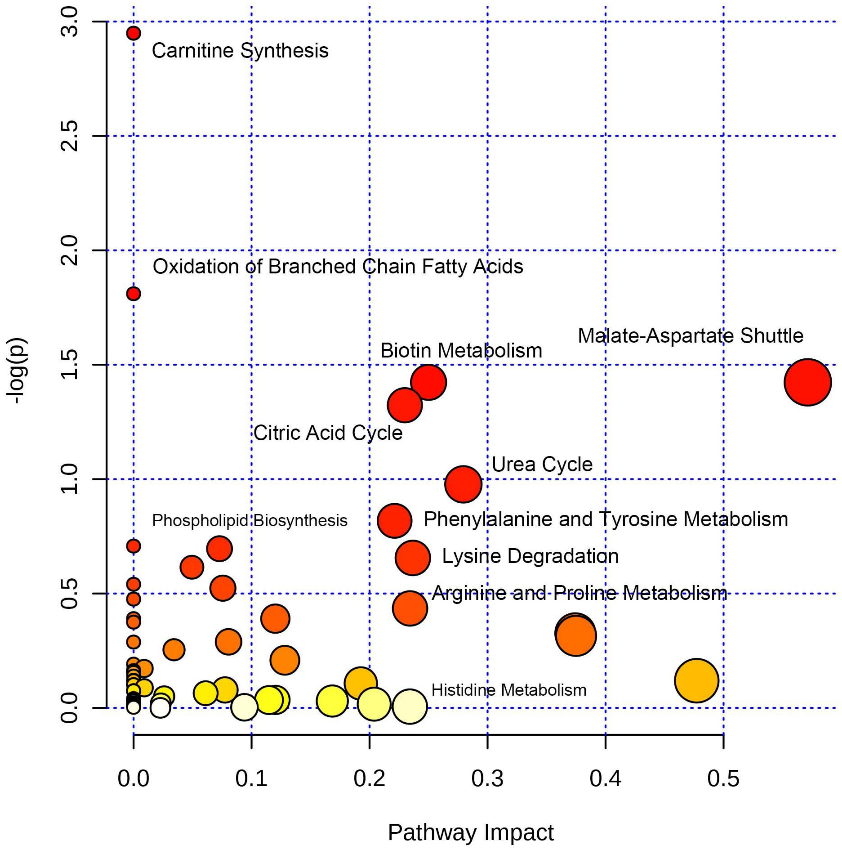

Fig. S2. Pathway enrichment analysis of identified metabolites.

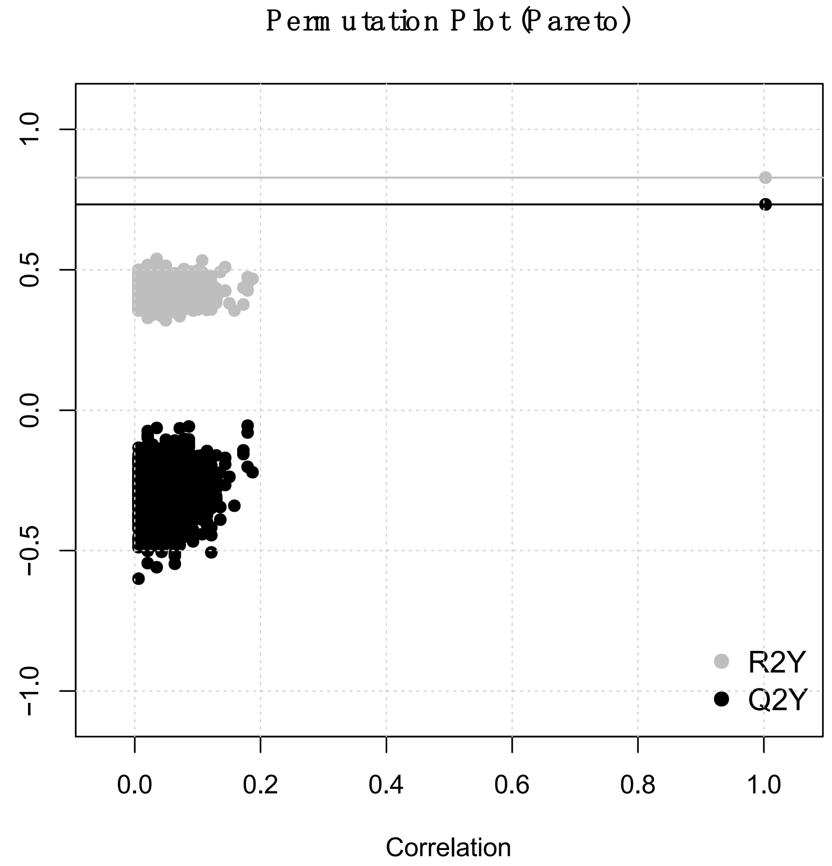

Fig. S3. The permutation tests (n = 1000) of PLS-DA model with pareto scaling.

Reference

Luan, H., *et al.* statTarget: A streamlined tool for signal drift correction and interpretations of quantitative mass spectrometry-based omics data. *Anal Chim Acta* 2018;1036:66-72.

Luan, H., *et al.* Comprehensive urinary metabolomic profiling and identification of potential noninvasive marker for idiopathic Parkinson's disease. *Sci Rep* 2015;5:13888.
